# Swipes and Saves: A Taxonomy of Factors Influencing Aesthetic Assessments and Perceived Beauty of Mobile Phone Photographs

**DOI:** 10.3389/fpsyg.2022.786977

**Published:** 2022-02-28

**Authors:** Helmut Leder, Jussi Hakala, Veli-Tapani Peltoketo, Christian Valuch, Matthew Pelowski

**Affiliations:** ^1^Department of Cognition, Emotion, and Methods in Psychology, Faculty of Psychology, University of Vienna, Vienna, Austria; ^2^Huawei Technologies Oy (Finland) Co. Ltd, Tampere, Finland

**Keywords:** photography, image aesthetics, individual differences, picture quality, contextual variables, object genre, user-centered model, smartphone camera

## Abstract

Digital images taken by mobile phones are the most frequent class of images created today. Due to their omnipresence and the many ways they are encountered, they require a specific focus in research. However, to date, there is no systematic compilation of the various factors that may determine our evaluations of such images, and thus no explanation of how users select and identify relatively “better” or “worse” photos. Here, we propose a theoretical taxonomy of factors influencing the aesthetic appeal of mobile phone photographs. Beyond addressing relatively basic/universal image characteristics, perhaps more related to fast (bottom-up) perceptual processing of an image, we also consider factors involved in the slower (top-down) re-appraisal or deepened aesthetic appreciation of an image. We span this taxonomy across specific types of picture genres commonly taken—portraits of other people, selfies, scenes and food. We also discuss the variety of goals, uses, and contextual aspects of users of mobile phone photography. As a working hypothesis, we propose that two main decisions are often made with mobile phone photographs: (1) Users assess images at a first glance—by swiping through a stack of images—focusing on visual aspects that might be decisive to classify them from “low quality” (too dark, out of focus) to “acceptable” to, in rare cases, “an exceptionally beautiful picture.” (2) Users make more deliberate decisions regarding one’s “favorite” picture or the desire to preserve or share a picture with others, which are presumably tied to aspects such as content, framing, but also culture or personality, which have largely been overlooked in empirical research on perception of photographs. In sum, the present review provides an overview of current focal areas and gaps in research and offers a working foundation for upcoming research on the perception of mobile phone photographs as well as future developments in the fields of image recording and sharing technology.

## Introduction

In today’s world, there are many millions of images taken every day. Most of these are taken by mobile phones. It is estimated that, in 2020, about 6 billion users took 1.4 trillion photos with their mobile devices.^1^ The mobile phone is our go-to device for documenting trips, social encounters, and important events such as weddings. It is the everyday camera for recording domestic events and memorable objects, fashion, pets or food. With the rise of social media and digital communication and the Internet, mobile photos are also a main means by which we record, curate, and communicate ourselves. Perhaps because of this prevalence of images, the camera has become one of the most emphasized features of mobile phones, with rapid technological improvements in aspects such as lenses and image sensors as well as image processing aspects including filters, color, and light correction. While these aspects certainly contribute to the technical quality of an image, the aesthetic appeal of the image most probably depends also on many more factors and psychological dimensions that are usually not covered by engineers (cf. Keelan, 2002).

How do we choose a good photograph on our mobile phones? Let us say you are with your friends and want to show them images of your recent hiking trip, your family gathering, a fancy dinner. What features make you think, “I really want to share *this* one?” Let us say you are handed another friend’s phone. When you are flipping through various pictures, what makes you stop and say “Wow, this is beautiful!?” “That is a great shot!.” Despite the prevalence of digital photos and phones in our lives, there is a lack of a systematic discussion of not only image-related, but also context- und user-related variables that affect our aesthetic responses to this domain. This leaves us without a means of explaining, anticipating, and empirically assessing the most basic of questions regarding our evaluations and selection decisions with mobile phone photographs—a current knowledge gap that both impacts our basic understanding of this medium, its relation to culture and communications, and the many pragmatic economic and social aspects involving one of the increasingly most basic features of our modern lives—mobile phone technology, and our digital existence. It is necessary to systematically investigate the factors affecting image beauty, from a psychological—perceiver—viewpoint and develop a wholistic model of perceived image beauty which goes beyond traditional image quality metrics. The latter typically focus on image-related properties such as resolution, dynamic range, noise, blurriness, or various visual artifacts (e.g., Zhai and Min, 2020; Duanmu et al., 2021; Tade and Vyas, 2021). However, in many cases, an image will not be perceived as aesthetically appealing or beautiful only for its lack of technical degradations. To understand the aesthetic appeal of images, it is necessary to move beyond image-related variables and take a broader set of contextual variables into account.

### Present Paper

The goal of this paper is to provide a systematic framework for understanding the aesthetic responses to photographs taken with mobile phones (hereafter Mobile Phone Photographs, MPP). We approach this question from the perspective of research in empirical aesthetics and visual preference^2^ (Leder and Nadal, 2014; Pelowski et al., 2017). Past models on art/aesthetics, especially in conjunction with empirical work done to consider basic features of visual processing and appreciation (color, contrast, complexity, etc.) provide an important basis for the present discussion. Here, we connect these models explicitly to the mobile photography domain. We start with a range of image-related factors such as color, or contrast. We also consider more intermediate processing-related factors such as symmetry or composition. We combine our approach with a consideration of the influences of interindividual, and cultural-context related sources of variation, motivations, and expectations—the latter factors of which might show key interactions and differences in terms of specific image factors when choosing photos. To make this maximally applicable to pragmatic discussions of how photos are used, we especially account for specific photo genres (i.e., contents), which represent the main types of pictures currently taken. Along with our review, we propose a first taxonomy of the variables that are important for the aesthetic evaluation of photographs in general, and MPP, more specifically.

### A Working Framework for Considering Mobile Phone Photographs

**Figure 1:**
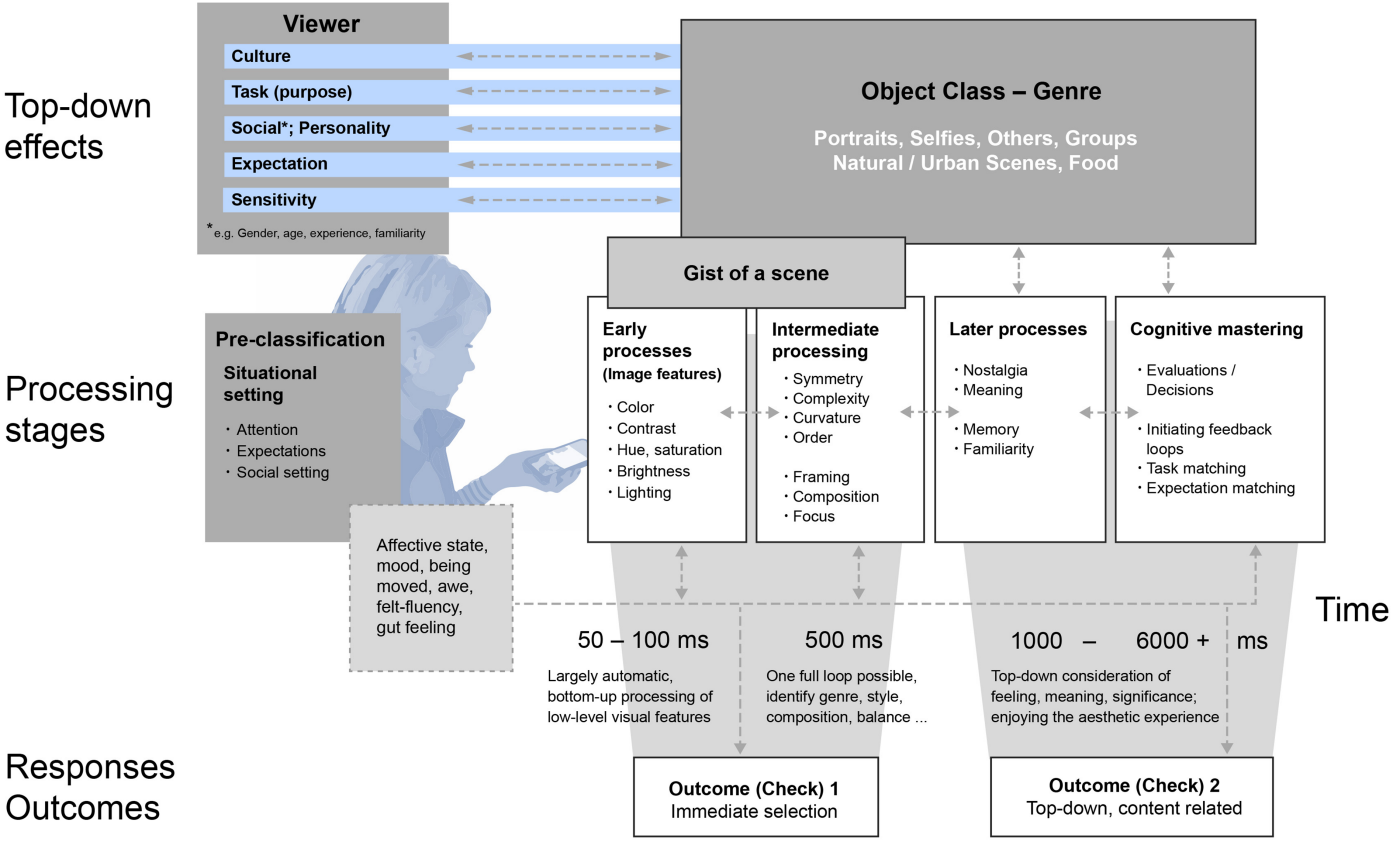
Main processing stages, influencing variables and two distinctive outcomes regarding beauty of mobile phone photographs.

Figure 1 shows the main components of our approach, which is inspired by previous aesthetic processing models of Leder et al. (2004) and Leder and Nadal (2014). In our framework, the individual user of the phone is in the center. On the left, we depict aspects that an individual brings to an encounter, involving specific expectations depending on the situation (i.e., I am expecting to see a snapshot, to select a photo for a specific purpose, to see something beautiful, or even artistry, etc.). We suggest that the photo genre (which objects are depicted) could be rather important in predicting which aspects might be considered during assessments of image beauty. The user himself is also a source of variation—as our sense of beauty also reflects previous experiences and specific taste (Leder et al., 2004) that might largely differ due to cultural backgrounds and other sources for differences in aesthetic standards.

Along four temporarily ordered processing stages, we have listed contextual aspects that we assume play an important role influencing two outcome checks which could be key to photo processing. This marks a departure from many models, which tend to flow toward only one (or a collection of rather equally weighted) outputs. Here, we are instead proposing an initial “quick” outcome check 1, and a longer or “secondary” outcome check 2 whereby viewers might decide on an image. Of course, we do not suggest that these different aesthetic experiences are determined by clearly distinctive, strictly linear, sequential processing stages (Campagna et al., 2020); however, we use this classification to allow a systematic assignment of features deemed particularly relevant for the outcomes. Specifically, we distinguish early visual processing, followed by an intermediate processing level, with more explicit top-down integration with memory where we also classify content—that is, object class or genre. This is followed by a “cognitive mastering” stage that was originally conceptualized in art processing models (Leder et al., 2004; see Belke, 2020). To accomplish a decision on the later level, the cognitive and affective processing at early stages is followed by the potential to essentially continue engagement, requiring re-looping through the model, toward a more involved, overtly top-down assessment. This requires a more time-consuming check of various image features, to refresh memories or allow to repeat pleasurable moments of image beauty. However, these processes often integrate schema for what makes a beautiful image of a certain genre and can have highly idiosyncratic, meaningful, and sometimes self-relevant elements (see Pelowski et al., 2017).

#### From Fast Preferences to Deeper Aesthetic Episodes

By varying the time provided to experience an image, it has been routinely shown that certain factors can be of more or less importance in evaluations, presumably as these may be attended to in different stages of the processing sequence (Leder et al., 2004; Pelowski et al., 2020). When considering visual artworks, we previously argued that to move through one loop in the basic model takes about 500 milliseconds which would align with the initial “gist” outcome suggested above. Importantly, a gist of a scene contains information about objects, if they are visually distinct or salient, and thus can activate schemata that also refer to specific objects, for example, that an urban scene contains houses, or that a supermarket scene contains shelves. Further studies have shown that we then might go through subsequent re-processing or re-looping through the model. As a general minimal time for this, previous papers have argued that about 6 s mark the point at which initial appraisals tend to become more robust and less impacted by specific evaluation durations. Similarly, emotional responses might be instantaneous and direct, but also might develop over time, with sudden, unexpected states of surprise and insight (Pelowski et al., 2017). While beauty can be experienced on a short time scale (e.g., Bachmann and Vipper, 1983), deep, absorbing aesthetic experiences surely need time to unfold their full power (Pelowski et al., 2017) and mere extension in time allows for an aesthetic experience to arise.^3^ Accordingly, the fast perception of beauty and slower, more content-focused processing can reflect partly independent modes of processing (cf. Redies, 2015).

Bar et al. (2006) demonstrated that preference judgments can be made within milliseconds of image exposure (see also Todorov and Porter, 2014). Bachmann and Vipper (1983) replicated and extended the findings by Cupchik and Berlyne (1979) that people could not only differentiate art styles based on collative properties, but that this differentiation already seemed to be apparent at very short presentation times in the range of milliseconds. Locher et al. (2007) argued that pictorial properties of an image (e.g., symmetry and complexity) can be perceived well within 100 ms, including a gist of an image comprising expressive aspects and aesthetic judgments. Augustin et al. (2008) found that processing of content in artworks (what is depicted) occurs as fast as 10 ms and is already strongly developed after presentations of around 50 ms, as is style. Similarly, Verhavert et al. (2017) showed that beauty judgments increased from 10, 20, 100 ms, but were available very fast, and might be based on low-level sensory visual information available within the first 40 ms. Thus, within 100 ms, a picture is probably already “understood” in the sense that observers seem to comprehend quite a lot of visual information, but a delay of a few hundred ms is required for the picture to be consolidated in memory. When consolidated, conceptual gist can be represented as a verbal description of the scene image. This seems relevant for fast swiping techniques, where micro decisions might frequently be made, but not necessarily remembered.

With increasing presentation time, as individuals can attend more to contextual aspects or cycle through the model attending to different features, the image becomes more differentiated and we may find differing types of interpretations and changing decisions (Bachmann and Vipper, 1983). As Belke (2020, p. 3) proposed, “appreciating art may be inextricably linked to an epistemic orientation that genuinely drives object-processing beyond habitual recognition-routines toward a higher reflective-level.” Although this epistemic orientation might sometimes take place in more mundane MPP, the everyday demands of delivering, illustrating, or memorizing events, serve goals that are distinctively different from typical encounters with artworks. Menninghaus et al. (2019), regarding aesthetic emotions, stated “aesthetic emotions are typically sought and savored for their own sake, with subjectively felt intensity and/or emotional arousal being rewards in their own right” and that they “entail motivational approach and avoidance tendencies, specifically, tendencies toward prolonged, repeated, or interrupted exposure and wanting to possess aesthetically pleasing objects” (p. 171).

#### Fast and Slow Decisions in Mobile Phone Photography

Following on this idea of fast decisions (Outcome Check 1), the use of mobile phones creates a situation in which fast and direct aesthetic responses (“swiping” or “giving likes”; cf. Thömmes and Hübner, 2020) are frequent and have become inherently part of the medium use. Related to aesthetic quality, evaluations of motif qualities such as “nice” or “hot” often are made so quickly that they are presumably based on some fast-accessible features of early vision, apparently associated with beauty, or their relative variation from image to image. For MPP, we suggest an interplay of two main kinds the processing of image features, that can be measured (e.g., captured through image statistics), as well as more intermediate, subjectively evaluated features which are analyzed very quickly, processed in interaction with the depicted topic (“explicit classification”) or genre, that also are seen very quickly, but their aesthetic evaluation might follow later. For example, the distinction between “this is a beautiful image” from “this is a beautiful image of you!” where the latter results from an explicit match between the schema of a photographed person, while that former might be implicit, more superficial. Therefore, we assume fast, sensory processing—while a stage in its own rite—provides the basis for later processes. We suggest that the later Outcome Check 2 usually has a higher stage of conscious awareness and that one’s decisions can be more explicitly articulated. On this processing level, a kind of matching between several variables is made—with expectation, with the prototypical set of images associated with an object genre; with the momentary goal and task. The feedback loops in Figure 1 illustrate some of the many possible paths of information processing and image feature combinations that are factored in when an MPP is assessed.

Naturally, many of the variables discussed in the present review are not restricted to MPP but should apply to the aesthetic appreciation of photographs more generally. Nevertheless, we chose to center our discussion around MPP because of the way these images are produced, selected, and shared with others. While the vast majority of MPP users lack training in photography, their devices make it easy to record pictures that are more or less flawless from an image quality perspective. Selecting the best pictures through swiping and deciding on which ones they wish to share with their friends online makes it an interactive and social experience. Also, swiping to select the best photographs on one’s own phone is similar to browsing through a stream of images on social networks such as Instagram. These aspects of MPP, together with the fact that some of the most shared genres of images (such as selfies or food images) are frequently produced with mobile phones, make it, in our view, a unique domain of study.

## Variables Affecting Image Aesthetics of Mobile Phone Photographs

### The Image: Early Visual Processing and Fast Preferences Based on Low-Level Image Features

#### Low-Level Perceptual Features

In empirical aesthetics, various psychologically relevant features have been studied in respect to image beauty and preference. Due to the scope of this review, we do not discuss in detail computational approaches that relate specific image properties to aesthetic judgments (see Brachmann and Redies, 2017, for a comprehensive overview on this topic). In our discussion we focus on the psychologically meaningful variables, and rather neglect the merely technical descriptors (Graham et al., 2016) that usually do not yet reveal a clear relationship to processes involved in image aestheticisms, although they are apparently related (Lyssenko et al., 2016; Mayer and Landwehr, 2018; Sidhu et al., 2018).

Some of the visual features have also been discussed as candidates for universal basics of visual aesthetics and beauty preferences. For different features, there are different underlying theoretical concepts that are often used to explain why they are preferred. For example, natural scene statistics are thought to elicit preferences due to their correspondence with naturally occurring patterns (Graham and Redies, 2010; Spehar and Taylor, 2013; Kardan et al., 2015; Graham et al., 2016). Some features are associated with learning and cultural imprint, such as certain color preferences; others with biologically, evolutionary determined positivity (such as familiarity and resulting processing fluency, Winkielman et al., 2003; Forster et al., 2013), or as a result of some correspondences to neuro-cognitive-affective features of the human visual processing systems (e.g., symmetry, Bertamini et al., 2018). Nevertheless, there is a clear need for more studies combining the very early visual image features to learn how these interact and eventually provide the necessary information to weight them in respect to aesthetic preferences. For example, Tinio et al. (2020) studied the separated and combined effects of contrast, sharpness, and visual grain degradations on aesthetic judgments of photographs depicting natural and human-made scenes. Oren et al. (2020) stated that their “conclusions call for future studies using multiple item types and various measurement methods for estimating value in order to modify current theories and construct a unifying framework regarding the relationship between low-level visual features and choice” (p. 1).

#### Perception and Preferences for Color and Brightness

Color, psychologically, is the sensation in our perception resulting from different photoreceptor activations, and their combinations, in relation to the overall distribution of light in the environment. Our visual system is very much attuned to recognize color, to an amount that Chaparro et al. (1993) argued “[c]olour is what the eye sees best.” Often, color is conceptualized as having three dimensions, hue (the wavelength), saturation (intensity, purity), and brightness (Specker et al., 2018). Although we tend to give different colors categorical names and colors are powerful features to identify objects (e.g., yellow is *the* determining feature for banana), these categories are culturally and language-dependent (Davidoff et al., 1999). However, as Convey et al. (2020) stressed, it seems that all cultures agree that black and white are the two most fundamental color categories.

Nascimento et al. (2017) found that viewers prefer the original colors in abstract paintings over rotated color versions, and found that artists’ color palettes throughout different epochs were dominated by the yellow-red range of the spectrum (cf. Altmann et al., 2021, for related results). There has been a long tradition to believe that (all or at least most) people appreciate certain colors more than others, but there is also strong evidence that color preference has a lot to do with cultural background providing associations and a history of individual learning. Palmer and Schloss (2010) show that people liked those colors which (measured from independent samples) were associated with objects they like (e.g., blue skies) while they dislike colors that are strongly associated with objects they dislike (e.g., brown). Since these associations may vary, this might explain why color preferences are not universally shared (Taylor et al., 2013). Jonauskaite et al. (2020) found that girls choose pink or purple as their favorite hue more often than boys do and that the most common favorite hue in girls and boys was blue. Moreover, in adults, pink never was the favorite hue; instead, it was blue.

People often prefer saturated colors, and use filters that saturate colors of the whole image or image parts. Skelton and Franklin (2020) reported that when colors were highly saturated, infants look longer at colors that adults prefer, and infant looking time and adult preference were highest for blue hues and are lowest for green/yellow hues. Reymond et al. (2020) argued that digital reproduction of artworks can vary greatly in saturation; however in an empirical study where color saturation was manipulated, this did not exert major influences on liking. Specker et al. (2018, p. 48) argued that variation in associations connected to hue are often due to cultural variation and represent influences of language on color discrimination (Winawer et al., 2007; Regier and Kay, 2009), cultural differences in affective meaning, as well as associations related to different hues (Saito, 1996). Probably, if two images differ in hue and saturation, the more saturated version is often preferred, but presumably only when the variables do not exceed a level of “naturalness” which could result in a Kitsch version (see Pelowski et al., 2020). This view is supported by a study of Nascimento et al. (2021) who found that more natural color combinations are also associated with stronger aesthetic preferences.

Going beyond specific color properties, brightness is generally associated with positivity—and therefore is preferred consistently with a variety of stimuli. Specker et al. (2018) concluded that “brightness seems to be associated with positivity across cultures” (p. 48). In relation to the emotional effect of color, Gao et al. (2007) even concluded that influences of hue and cultural background are limited, whereas brightness could be seen as a universally important influence. Specker et al. (2018) explain this with our visual system employing two pathways, a chromatic one that processes color contrasts and an achromatic one that processes luminance contrasts (for a detailed discussion and evidence see Gegenfurtner and Kiper, 2003).

#### Lighting Conditions/Hue

An aspect related to color perception is *color temperature*, as a means of quantifying the color impression of a light source. Skelton and Franklin (2020) argued that the colors which are more generally liked tend to be colder (blue) rather than warmer (yellow). In sum, the available literature suggests considerable interindividual differences regarding the perceived beauty of colors.

Naturally, the distribution of light changes over the day, and images representing the light conditions at dawn are considered particularly beautiful. MPP are often taken indoor, under artificial lighting conditions. Another way of considering this is as hue, or the general color temperature of objects, often as a result of the temperature of the lighting. For humans as a visual species, lighting has been a key aspect of human perception and impacts aesthetic qualities (Pelowski et al., 2019 for review). In general, many theoretical and also art-related discussions (e.g., considering the best light color temperature and conditions for maximizing displayed art’s beauty) have suggested target temperatures such as that around natural daylight (e.g., 5,500 K) or a relatively warmer interior (3,000 K). However, in reality, emerging empirical studies show a wide range of preferences and differences, although still relatively within the above ranges, most probably based on contextual variables—such as the exact study design or the nature of the illuminated objects (Pelowski et al., 2019). (For other art studies see also Nascimento and Masuda, 2014; or Pinto et al., 2008; for television displays, see Sproson, 1983). For MPP, we can assume that a wholistic image manipulation toward a natural light temperature might positively affect all images. The beauty of images could also be improved through lighting, either by choosing different filters that change the range of light on a digital image, for example, removing disturbing shadows (Zhang et al., 2020a), or by changing the light in the environment; the latter is an important feature, particularly for portrait photographers. For instance, light direction in a portrait changes the appearance of a person quite dramatically (Marković et al., 2019), as does the softness of the shadows which varies with light source size.

#### Contrast and Sharpness of Images

Psychologically, the concepts of contrast, and image sharpness are often closely related. Both have well-studied effects on image beauty. Contrast is often used as a manipulation of visual fluency—and, as a consequence, subjectively perceived fluency (Forster et al., 2013) where it produces reliable effects on preference (Reber et al., 1998). In these studies, researchers also have manipulated perceptual fluency through different degrees of figure-ground contrast and found that participants liked the same image more with higher figure-ground contrast. van Dongen and Zijlmans (2017) found consistent preferences for high over low contrast versions, independent of cultural or social background of the perceiver. Findings also suggest that relative sharpness of an image usually is appreciated over blurriness. Leder (2001) showed, much as with contrast, that more fluently made versions of sharper pastel paintings were preferred, but that a short period of familiarization could also change the preference toward the less sharp-contrast versions. These findings are in contrast with the aesthetic practice to smoothen images to make them more attractive and beautiful, as in “soft glam” effects from the 1970s, which may be more important with people photos. Filters producing softened edges can also smooth skin and make faces appear more averaged (Langlois and Roggman, 1990) which could conceal less attractive features in faces (Leder et al., 2017). Selective focus also helps segmenting a photograph through bringing some parts into focus while deliberately defocusing others. In MPP, such effects are implemented using advanced image processing techniques that blur parts of an image to simulate “bokeh” effects (Ignatov et al., 2020).

#### Basic Shape Features—Curvature

Low-level features of early vision also comprise features of shape, although the distinction from more intermediate features might be discussed. The most frequently studied shape feature has been curvature, which has even been discussed as a possible universal feature of preference in humans (e.g., Munar et al., 2015; Palumbo et al., 2015). Bar and Neta (2006) showed that under very brief presentation times (84 ms), participants preferred various objects, furniture, household objects, and abstract patterns, with curved contours, and suggested that this would be in accordance with sharp shapes being more associated with fear (Bar and Neta, 2007). Leder et al. (2011) also showed that this effect interacted with object’s valence and was mainly found for neutral and positively valanced objects. Thus, curvature affects beauty of many objects, geometric forms, household objects, furniture, or car interiors (Leder and Carbon, 2005), and this effect persists even when controlling for symmetry, prototypicality, and balance. The positive effect of curvature on beauty has also been shown in the appreciation of curved interior spaces (Vartanian, et al., 2013b). Vartanian et al. (2013b) and Thömmes and Hübner (2018) found that curvature was positively valued in architectural photographs.

### The Image: Intermediate Visual Properties

On an intermediate level of image processing, a more wholistic impression is built at a similar temporal scale as early features are processed. Berlyne’s empirical works with visual pattern revealed the importance of variables such as complexity, novelty, order, or ambiguity which he termed “collative” (Berlyne, 1970). Cupchik and Berlyne (1979) presented two studies, employing reproductions of paintings and artificial patterns that varied in collative properties and found that perceivers could discriminate these properties after only a single glance (50 ms), and of course after much longer presentation times (500 and 5,000 ms).

However, if a scene is initially processed as a single entity, then, what is the nature of this entity? An alternative approach to gist representation (Oliva and Torralba, 2001, p. 255) takes “advantage of the regularities found in the statistical distribution of image properties when considering a specific scene category (e.g., a highway must afford speed, so ground is a flat surface stretching to the horizon). Along these lines, perceptual and conceptual representations of gist could be initiated without processing object information.” Oliva and Torralba (2001) argued that, since real-world scenes are arranged in three-dimensional space, fast scene recognition could be based on image properties that are diagnostic of the space the scene subtends. They “found that eight perceptual dimensions capture most of the three-dimensional structures of real-world scenes (naturalness, openness, perspective or expansion, size or roughness, ruggedness, mean depth, symmetry, and complexity).” Research regarding variables discussed in this section suggests that each of them can affect beauty and preferences, but there are many open questions, such as, whether and how these features interact and whether they affect image beauty independent from each other.

#### Symmetry

Symmetry, as a design principle, has a long research tradition in aesthetics and biology and has even been considered a super-principle of beauty that is also associated with positive valence and arousal (Bertamini et al., 2013, 2018, 2019). Leder et al. (2019, p. 2) stated that “Researchers have repeatedly shown symmetry’s central role in preference and beauty judgments of visual stimuli, including biological entities such as faces.” It has been argued that the preference for symmetry in faces and bodies emerged as a kind of gold-standard for beauty indicating favorable features for mating, such as good health, stable development, and good genes (Thornhill and Møller, 1997). However, symmetry is also often preferred in meaningless, abstract patterns (Jacobsen and Höfel, 2002; Tinio and Leder, 2009b; Gartus and Leder, 2013) and can be seen as being processed fluently and efficiently (Reber et al., 2004). However, Sadr and Krowicki (2019) also presented challenging results when they found that half-occluded faces that lose all symmetry are found more attractive than their complete versions, and Leder et al. (2019) showed that art experts did not prefer symmetry. Nevertheless, vertical symmetry might be considered particularly beautiful in the case of faces and bodies, and perhaps also food arrangements, whereas horizontal mirror symmetry might play a role especially in landscapes.

#### Complexity

Multiple studies have found that more complex patterns are generally preferred over simple patterns (Hekkert and van Wieringen, 1990; Jacobsen and Höfel, 2002; Tinio and Leder, 2009b). But with increasingly more complex material the findings also become more ambiguous. For example, individuals can also differ with respect to whether they prefer simpler or more complex patterns. Averaging across individuals can give the impression of an optimal degree of intermediate complexity that does not reflect specific individual preferences (Güclütürk et al., 2016; Spehar et al., 2016). Nadal et al. (2010) presented a comprehensive approach, studying artworks and images, and measured various evaluations of complexity, and related them to beauty. Surprisingly, they found no evidence for a systematic effect of complexity on beauty but could distinguish three types of complexity; related to the quantity and variety of elements, spatial arrangement, and to asymmetry, which affected beauty differently. Complexity also differs between artworks and photographs of environmental scenes, relying on different image variables (Marin and Leder, 2013). Another important aspect of complexity is the top-down, knowledge-related processing of meaning which reduces and determines semantic complexity very fast (Commare et al., 2018). This is more relevant in ambiguous images, such as artworks, but somehow also affects all images that have encrypted meaning. In MPP, the role of complexity needs further study, including the roles of task and class of object.

### The Image as a Result of Decisions Taken by the Photographer

#### Order and Balance

Closely related to complexity, beyond the perception of isolated stimulus properties, the way elements in images are spatially arranged has effects through order and balance. Locher and Nagy (1996) showed that quite subtle differences in balance could be seen after 100 ms presentation. Wilson and Chatterjee (2005) stated that perceivers prefer movement in images from left to right, and have preferences due to the location of the most salient object. They also argued that preferences for balance are likely related to pre-attentive visual processing, because people are sensitive to balance “at a single glance.” Friedenberg (2020) studied preferences for different ordering of elements, that create texture and found that viewers preferred patterns through which the eye can travel unimpeded. Several features including collinearity, symmetry, and simplicity help to explain the data and are associated with the processing fluency model. Arnheim (1971), as a proponent of Gestalt theory, would also have suggested to discuss the kind of representations that guide fast responses of “this feels right” as guided by Prägnanz and “Good Gestalt” laws developed in this school. Allesch (2016) rightfully concluded that the typical approach claims that the perceptual impression of the whole dominates, and this wholistic representation is what allows the analytical consideration of visual details. For a better understanding of the interplay between order, balance, and complexity it is also necessary to take individual differences into account (for a discussion, see Van Geert and Wagemans, 2020).

#### Cropping, Framing, Composition, and Focus

Cropping, Framing, Composition, and Focus are the result of decisions in the moment of taking the picture, but also post-processing the image (see McManus and Stöver, 2014). These characteristics are kind of an interaction between image features and person features that rely on decisions and actions of the photographer. Each picture taken also represents a decision to select a cutout of the diversity of possible cutouts. Very little is known about such cropping decisions, and how they are made, or even how they affect the beauty of the resulting image (cf. McManus et al., 2011). In one of the few examples, Abeln et al. (2016) implemented a study on details selected and details avoided during cropping and found that, on average, selected details contain regions of higher visual saliency than avoided details, and that the center of mass in the chosen framing was close to the geometrical center. The authors stressed that the kind of cropping they studied belongs to the variables that bottom-up determine aesthetic evaluations of photograph images. In terms of MPP, the difference in cropping might be particularly salient in the Outcome 1—fast decisions, when swiping through a series of images of the same topic. Moreover, background-foreground or grouping and putting-together of objects might be chosen by the photographer. Positioning objects in an image can be used to bring things literally into perspective. Beyond the more general term “cropping,” each camera allows to turn the angle or choose between the format options Landscape/Portrait. As the names suggest there is an implicit assumption that these formats would be better suited for either kind of genre, portrait or landscape.

The theory of photography has developed sets of rules that enable “good” compositions (see, e.g., Feininger, 1973)—the rule of thirds, the golden ratio, triangular composition, but also central perspective are examples of creating aesthetically pleasing images. There is evidence from professional teaching, art history, and image studies, demonstrating how perspective determines beauty but proper empirical studies are rare. Even regarding the golden ratio, evidence for a positive influence on aesthetic preference is ambiguous (Stieger and Swami, 2015). In a recent eye tracking study, Torabi and Teeravarunyou (2021) found that while experts with a photography background find images that follow the rule of thirds overall more interesting, novices were not as sensitive to this type of general guideline. On the other hand, Locher (2003) studied what he called “visual rightness” and found evidence for the view that visually right (i.e., “good”) compositions have efficient structural organizations that are visually salient to viewers who lack formal training in the visual arts. However, how these effects translate into meaningful psychological states, when looking at photographs, is a largely open question.

In the human eye, the lens produces an area of sharpness which corresponds to the focal plane, usually in the very center of the visual field while the spatial resolution in the visual periphery is drastically reduced. Those details of an image that are directly gazed at are the ones that are later remembered and often revisited with the eyes when a familiar image is viewed again (Valuch et al., 2013). In contrast to the human eye, the focal plane in a photograph is decided by the photographer and does not necessarily lie in the center of the image. The area in focus usually has the objectively highest visual resolution while other peripheral image parts lack this focus independent of the attention or focus of the perceiver (see Zhang et al., 2020b). How much of an image is in focus also depends on optical variables, such as the aperature and the focal length of the lens relative to the size of the image sensor. This assigns the procedure of focusing a special status in image production and perception, and thus, also in aesthetic appreciation. Interestingly, digital cameras allow focus areas in images that are much bigger than what the human eye could perceive with high acuity. However, it remains unclear how “oversharp” images shape perceiver’s expectations of how certain types of images should look like, or whether this feature could moderate interestingness—as an important ingredient of aesthetic quality in images (Leder et al., 2004).

Mobile phone images can vary along all the discussed features, and, in early vision, these can prime preferences—especially in a sequence of other images. Importantly, a gist of a scene contains information about objects, if they are visually distinct or salient, or activate schemata that also refer to specific objects, for example, an urban scene contains houses, a supermarket scene contains shelves. Thus, the presence of specific objects is a strong determinant of different schemata for beauty (Augustin et al., 2012).

## The Object: Content and Genre—What Is a Photo “of” and What Is it “for”?

On mobile phones, we assume, the genre could be the most relevant level of aesthetic decisions. Not only do genres differ in the amount to which users expect them to be beautiful, they may vary in whether beauty is even a relevant dimension (e.g., selfies might look for this; snapshots of events might not have to be beautiful) and even ugly or shocking content that would not be rated as beautiful can constitute powerful or memorable images. Generally, when humans view images, pictorial content plays a decisive role in evoking emotions (Redies et al., 2020), objects attract attention (Einhäuser et al., 2008), and determine what is aesthetically preferred. People differ in what kind of images they regularly look at or produce themselves. Research showed that the strength of individual preferences for specific genres or categories are reflected in the activity in brain regions associated with object valuation such as the ventromedial prefrontal cortex (Lopez-Persem et al., 2016). Moreover, beyond the above-described gist of scenes, at least after about 150 ms, correlates of high-level properties of the image can be observed in ERPs, for example, for faces (Jeffreys, 1996) and other objects (Rossion et al., 2000).

We assume that the object genre provides a kind of classification regarding the criteria of beauty of images discussed so far. Therefore, in the next paragraphs we provide very brief summaries of features that have been discussed specifically to increase or decrease beauty of each kind of object. Specific object genres seem to be particularly prevalent in MPP, which is evident in the high frequency with which these types of images occur among the huge number of images taken with mobile phones on a daily basis (e.g., Hu et al., 2014). Based on this prior evidence, we chose the following classes of objects: portraits (of oneself and other people), or groups and “Groufies” which are Selfies including the photographer (in Hu et al., 2014 also called “friends”). As further interesting and frequent categories, we look at food images and (different from Hu et al., 2014) outdoor scenes of natural and built environments.

### Portraits

Facial attractiveness is a huge research topic, with thousands of papers studying what kind of faces and facial features people find attractive and beautiful. We can only give a brief overview here and focus on features that can be deemed particularly interesting for understanding beauty of faces in MPP. Faces in more complex images can be localized very quickly in complex natural scenes, at about 40 ms (Campagna et al., 2020). Pictures with faces attract more likes and comments on Instagram (Bakhshi et al., 2014). Facial beauty or attractiveness is a very pervasive factor in everyday life, affecting our social perception and interactions in various ways (Tsukiura and Cabeza, 2011). Looking at attractive faces is rewarding, and elicits positive emotions (Aharon et al., 2001; Gerger et al., 2011; Vartanian et al., 2013a; Hahn and Perrett, 2014), beautiful faces attract and bind attention (Valuch et al., 2015; Leder et al., 2016b; Mitrovic et al., 2016). Moreover, beautiful faces also have a different kind of visual power. For example, Nakamura and Kawabata (2018) presented evidence that facial attractiveness leads to faster conscious reports of faces that are initially invisible due to interocular suppression, which reflects differences in processing of beauty at a very early (and possibly unconscious) stage. However, while the aesthetic value of portraits is presumably correlated with face beauty, an artistic portrait can be beautifully crafted and aesthetically appealing when considered for more extended durations, even if the depicted face is not attractive (cf. Schulz and Hayn-Leichsenring, 2017).

#### Factors Associated With Face Beauty

Various factors have been discussed, to determine levels of beauty in faces (for reviews see Gangestad and Scheyd, 2005; Rhodes, 2006; Little, 2014). Among the most widely studied features is averageness as an average expression in size, location, texture, etc. of facial elements which is particularly attractive (Rhodes, 2006). Symmetry has a smaller effect on facial beauty and, was even considered a subordinate effect to averageness (Rhodes et al., 1999). However, most so-called “beautification” filters make use of some kind of averaging procedure (e.g., https://github.com/beholdergan/Beholder-GAN, see Diamant et al., 2019). Symmetry and averageness can be manipulated through makeup and by concealing elements that deviate from an ideal symmetrical or average face. Youthful features, with smooth skin, and gender prototypical appearances, are often found attractive. Consequently, coloring indicating fertility (in women)—reddish skin—also contribute to beauty (Jones et al., 2015). Gender prototypicality refers to features that distinguish male from female faces; or features that are particularly indicative of marked masculinity or femininity. Masculine male and feminine female faces are often judged as particularly beautiful. Moreover, a feature that might be more important in MPP than in any other field is cuteness, a feature of faces (Little, 2012)—and also of other objects, such as pets, or even fronts of small cars (Miesler et al., 2011).

Importantly, as humans are highly sociable, social aspects of face appearance also have a strong impact on attractiveness: Faces are more attractive when they smile, and aspects of a person that develop with social encounters such as familiarity, personality, pleasurable interactions, perhaps are often stronger than early extracted visual features. Specifically, in Kaisler and Leder et al. (2017), we found that social evaluations such as trustworthiness are unaffected by the aesthetic advantageous perspective of three-quarter views. Regarding deviations from the ideal beauty of images, especially on MPP, one has to mention the “red eye” effect, and unfavorable moments such as blinking—closed eyes, funny, or strange facial expressions. Thus, removing or concealing all kinds of negatively deviating features would improve the perceived beauty of faces in photographs (Leder et al., 2017; Sadr and Krowicki, 2019). A challenge for MPP could be to distinguish unfavorable features from “beauty spots” that are associated with depicted person’s identity and might even increase perceived attractiveness (cf. Springer et al., 2007).

#### Selfie and Self-Portrait

Probably the most typical for mobile phone images, are Selfies, self-portraits taken with a handheld camera directed at the own face, the type of images most commonly posted on social media. While their beauty depends on the same criteria as other face images, their aesthetic quality is also established by expectations and self-images of the photographer and the beholder, as they are expected to express the person, in appearance and beyond. There is some research related to the specific way selfies are taken, limited by the arm position and resulting in different orientations of the depicted face. Mobile phones usually include wide-angle selfie cameras which result in specific distortions of the face and body relations and sets this class of images also visually apart from more commonly used longer focal lengths in portrait photography (Cooper et al., 2012). Kalayeh et al. (2015) employed computer vision and machine learning techniques and found that shiny hair and smiles were among the most relevant features. Yeh and Lin (2014) estimated the aesthetic quality of a selfie based on the facial angle, and Schneider and Carbon (2017) used 3D head models to demonstrate that certain angles for making selfies are more favorable than others (e.g., that attractiveness was higher when the image had been taken from above left). There are also indications that selfie-posting could eventually even be regarded as a new online experience of sexual objectification that could affect goals and consequences of MPP (Zheng et al., 2019).

The rather new genre of Selfies demands interesting questions for the psychology of beauty: Whether people indeed employ the same criteria for judging beauty of their own faces as they do for faces of others, and to what amount this interacts with familiarity in general. One can also assume that the positive effects of smiles in portraits might (at least sometimes) be different in Selfies, or show stronger interactions with self-image (e.g., “I want to be seen as serious.”) and thus interact with task, purpose as well as with the personality of the photographer.

#### Other Persons (Family and Friends)

Although it is tempting to assume that the criteria for facial beauty generalizes to all kinds of face images, the distinction between self, familiar others, or unfamiliar faces might be relevant. While face beauty often aimed to study universal features, such as averageness, symmetry or apparent youth, and smiles, in familiar faces, the typicality of the image for the person might show strong interactions between identity and attractiveness. It can also be possible that familiar faces show a certain level of “hard wired—strongly associated level or value of beauty” (for example, “Ingrid Bergmann is pretty, Marty Feldmann rather less.”). However, recent studies rather show that such anchoring effects are more episodic, or image-bound (Goller et al., 2019). Moreover, the beauty of an unfamiliar face might serve different purposes, such as providing aesthetic pleasure, as artworks do, or information about aspects of beauty that play a lesser role in familiar faces (e.g., makeup, styling, or digital manipulation).

#### Group Portraits

Until now, there is little research on the topic of group portraits. There is no clear distinction in most research regarding “portrait” and “group.” Looking at an image of a person on screen already creates a kind of dyadic situation (Kaisler and Leder, 2016). However, very little is known about the criteria that people might apply to evaluate either as beautiful. Initial evidence that groups have their own standards of beauty comes from a very recent study by Brielmann et al. (2020) who concluded beauty is often intensely experienced in nature and that “research on aesthetic appreciation that claims relevance for everyday beauty experiences should include such social and nature-related experiences.”

When an observer looks at an image of two other people, many cues arise from the two perceived faces simultaneously. In a number of studies, we tested how the presence of another person in a scene affects the evaluation of the two people (Kaisler and Leder, 2016, 2017; Kaisler et al., 2020). Kaisler and Leder (2016) presented natural scenes with two faces showing different directions of gaze. Faces looking directly at the perceiver were rated as more attractive and as more trustworthy. Positive effects on evaluations were also found for ¾ views (Kaisler and Leder, 2017) and smiling faces (Kaisler et al., 2020). Together these findings show which features of another person present in an image affects social and aesthetic evaluations.

Group portraits also show interactions with the specific context and task; when used to document a team, as compared to a private picnic, different kinds of composition might be dominant: in the former a more evenly distributed arrangement of depicted persons, eventually higher symmetry; in the latter as more lively, less ordered arrangements might be found favorable. There is also a new genre, “Groufie,” which includes the photographer. Wang et al. (2020) specifically studied group photographs, considering opened-eye, gaze, smile, occluded faces, face orientation, facial blur, and character center. From a large dataset of group photographs they stated these „features perform well for categorizing professional photos and snapshots and predicting the distinction of multiple group photographs of diverse human states under the same scene” (p. 1). This kind of combination of genre-specific criteria and aesthetic appeal are good examples for a taxonomy of features that affect perceived beauty. For all kinds of group images, it remains an open question, whether the beauty of the image can be predicted by the beauty of the constituent elements, and if so, in which way these interact.

### Scenes

In psychological perception research and empirical aesthetics, scenes are a frequent topic of inquiry, and photographs of scenes, during the last decades, have been taken as representations of the “real world.” An often-made distinction is between nature scenes, the more classical topic of landscapes, and built environments. The latter can comprise street scenes, urban scenes, buildings, touristic views of city, as well as also sometimes scenes of human-made interiors. Most generally, natural scenes seem to be preferred over built environments (e.g., Biederman and Vessel, 2006). Aesthetic preferences for nature often are explained by evolutionary adaptations, as nature for long time was the habitat that shaped human aesthetic preferences. Accordingly, “naturalness,” for example of color reproduction, may be an important dimension for the judgment of real-world scene depictions (Yendrikhovskij et al., 1999). Important for MPP, this relation—nature versus human-made also depends on the image quality, and can therefore even be reversed (Tinio and Leder, 2009a). Moreover, as already discussed in respect to gist of a scenes, the affective (and probably aesthetic) evaluation of environmental scenes occurs very fast (e.g., Hietanen et al., 2007).

#### Natural Scenes

Natural scenes often are found particularly beautiful. Various stimulus properties are powerful determinants of preference for most observers (Kardan et al., 2015). For example, evolutionary approaches claim that natural features, such as green vegetation, or blue skies, and places to hide as well as hints toward water and food (all these correspond to a prototypical, survival-related appearance) are important factors for aesthetic evaluations (e.g., Kaplan, 1987). However, natural scenes contain a considerable amount of high-spatial frequency chromatic information that is not processed by the human visual system (Párraga et al., 1998). From a more cognitive perspective, the perceptual processing fluency associated with the image might also be of importance (e.g., Reber et al., 2004) which could be related to factors such as the clarity of the image (“high contrast”) but also familiar, natural distributions of complexity of certain features might serve as sources of aesthetic preference (cf. Steen, 2006).

#### Human-Made Scenes and Urban Environments

Preferences for urban scenes are manifold, they differ largely in content and show large interindividual varieties in aesthetic appeal (see Weber et al., 2008). What one person likes, might be highly disliked by another. This is possibly the case because preferences for human-made, built environments are strongly determined by variation and individuality (see Smith, 2014)—or conformity—in taste for buildings, certain architectural styles, and their fashion, personal associations, and memories (e.g., nostalgia), but also cultural imprint, as well as education level. Environmental psychology has delivered some general principles of beauty that also apply to preferences for built environments, such as complexity, unity in variety and curvatures, but also whether there is a dominant object, such as a church or a temple, as well as the ordering of all architectural elements; also, the presence of green, of plants, or of people in urban scenes will presumably affect how much they are liked. From an empirical aesthetics perspective, comparable to artworks (Leder et al., 2004), urban scenes largely differ in the style of the depicted buildings, and very little research studied the often-claimed “common” preferences for certain styles (e.g., older preferred to more modern facades).

There are estimates that on average, people in western cultures spend most of their time indoors (for Americans, even up to 90%; Klepeis et al., 2001). In psychology, only little research has focused on the features that determine aesthetic preferences for some indoor settings. A key challenge for this type of research is that, depending on current fashion trends, what is considered fashionable and “state of the art” among architects. Moreover, cultural differences also play a role, as interior design varies a lot depending on climate, social status, and generally cultural imprint. In a study regarding the preference for interior space combining evaluations and brain responses, Vartanian et al. (2013b) found that curvilinear interior spaces were judged beautiful. Moreover, neuroanatomically, when contemplating beauty, curvilinear contours are associated with increased activations of the anterior cingulate cortex, representing reward properties and object’s emotional salience. Pleasantness accounted for nearly 60% of the variance in beauty ratings. To investigate which kinds of city images people like, Zasina (2018) analyzed data from Instagram and found that urban images represented mostly aestheticized and picturesque places and objects in the urban environment.

One intriguing sub-genre of urban scenes (although also other genres, such as faces) are night images, taken under low light conditions. Due to technical advances, the pictures that can be taken at night using modern mobile phones are increasingly impressive in quality, and sometimes display luminance distributions similar to daylight images. The psychological expectation of what a photographed night scene should look like to be aesthetically appealing might require a very fine-tuned match. There might be a broader range of remembered elements, and perhaps more importantly, a wholistic impression, to which the image of the urban night scene should match. Here, a certain level of naturalness is probably required, as otherwise a kind of alienation might occur, caused by the mismatch of image features and expectations, maybe even feelings similar to “uncanny valley” (e.g., MacDorman and Chaatopadhyay, 2016) which could strongly undermine the aesthetic appeal of the image.

### Food

There are estimates that more than 62,000 new photos are shared globally each day under the hashtag “#foodporn” alone (Mejova et al., 2016). Although food and eating are among the basic activities that humans need for survival, the sheer amount of food pictures shared is surprising, and an example of how newly emerged media (mobile phones in combination with social media) create a new field of aesthetics that fascinates many thousands of people (Taylor and Keating, 2018). Oren et al. (2020) showed that when preference was tested for images of snacks, items with higher luminance (Milosavljevic et al., 2012) or higher saliency (Towal et al., 2013) were more likely to be chosen in forced choice tasks. Zellner et al. (2011) described how “visual properties of a food affect our expectations concerning its chemosensory qualities and also its hedonic value, for example. That clear beverages might be refreshing (Zellner and Durlach, 2003). Zellner et al. (2011) also demonstrated the positive effect of neat arrangements on a plate. Rowley and Spence (2018) found that plates of food were liked more when the food on the plate was horizontally and/or centrally arranged.

Beyond saturated colors, variables, such as glace and shininess might also increase the visual appeal of food, if they stress positively valued elements in the image (e.g., the smoothness or thickness of a sauce), see Spence and Piqueras-Fiszman (2014). Paakki et al. (2019) found that women were more interested in the aesthetics of food plates than men, and when asked about choice between differently colored lunch portions (color-manipulated pictures), most people preferred plates with a wide range of colors. Regarding associations elicited by beauty of food images, Hagen (2020) found, that “people judged prettier versions of the same food as healthier (e.g., more nutrients and less fat), despite equal perceived price” and stressed the danger of misleading beauty. Kakimori et al. (2016) proposed a support system to help amateurs to improve the beauty of their food images, to make the food look more delicious, by helping with camera tilt and composition.

Versace et al. (2018) studied the relationship between susceptibility to food-related cues (images) and food consumption, offering a contribution to the understanding the neurobiological basis of vulnerability to obesity based on cues from food images (but also see Spence et al., 2016 on visual hunger). Peng and Jemmott (2018) found that people tended to like (but also share images) when they contain tasty foods. However, aesthetic appeal and specific visual features, such as arousing colors and components of visual complexity, also were relevant.

As an interesting step to study the visual features that make food photos beautiful, Sheng et al. (2020) provided a Gourmet Photography Dataset, with 24,000 food images, from which 13,088 were assessed as positive and 10,912 as negative according to anonymous raters and expert evaluations (https://github.com/Openning07/GPA; also see Toet et al., 2019, for an alternative). They concluded that “There is an increasing amount of research into food images, because of its high value in commercial visual marketing.” (p. 2). In their study, Sheng et al. (2020) provided algorithms that learned to distinguish beautiful from non-beautiful food images. The authors also stressed the role of color in these evaluations and found that people agreed more on negative visual aesthetics than on positive aesthetic judgment.

## The User

Probably the most influential while less intensely studied element in the beauty of images, especially MPP, is the user—what the person brings into any aesthetic setting in terms of taste, learned associations, education, age, gender, or culture. While few studies give clear indications of how variation and features of the user affect beauty appeal of MPP, at least some indications can be found in the literature which could serve as a starting point for developing a more comprehensive theory. We distinguish these trait-like user variables, from the much more dynamic features of task, expectation, and situation. If the aesthetic sense is seen as a tool that evolved to foster good decisions, originally regarding more fundamental biological functions, such as mating, finding friends, or favorable habitats, the sense is probably a combination of general, universally established relationships between certain features and their appeal as beautiful. However, as the aesthetic sense is shaped trough experience it also must contain aspects that represent the beholder’s individual histories and are often described as personal, individual taste (Leder et al., 2016a).

### The Individual Aesthetic Taste and Aesthetic Sensitivity

To cite Korhonen (2019), “Since there is large deviation in personal opinions and aesthetic standards, the next challenge is to find the settings and post-processing techniques that fit to the individual users’ personal taste” (p. 1), with the goal to personalize the camera use. Regarding differences in aesthetic sensitivity, recently, Mitrovic et al. (2020) reported that in the VAST (Götz, 1985; an aesthetic sensitivity test where participants have to judge which of two images is more beautiful), the image that is preferred by participants is also viewed longer (also see Myszkowski and Storme, 2017), but that this effect occurred in line with participants’ subjective tastes, not with the pre-assigned “expert-suggested-favorites.” Thus, as with faces and other objects, longer looks at more beautiful objects rely on individual, subjective evaluations of beauty. However, there have also been other tasks suggesting similar preference pattern as the VAST (e.g., Stich et al., 2007). Corradi et al. (2020) introduced a new conception of aesthetic sensitivity defined as the extent to which someone’s aesthetic valuation is influenced by a given feature and revealed that people differ remarkably in the extent to which visual features influence their liking, which stresses the role of individual variation. Fenner (2020) argued that knowledge plays an important role for the development of aesthetic taste, at least for art. Nadal and Chatterjee (2019) explain that with “each encounter with an artwork neural networks are engaged that are modulated by context, expectations, emotional states, goals, and experience,” and that over time that repeated “encounters with art over the course of a lifetime lead people to develop personal preferences, as the network connections become strengthened in unique ways” (p. 1). Thus, encounters and familiarity are deemed important for any kind of taste, preference, and beauty (Leder, 2001; Reber et al., 2004; Vogel et al., 2020). Aleem et al. (2020) provided a step toward a general and unifying framework for understanding the various aspects involved in the formation of aesthetic values over time that also considers motivations and changes over time. Interest and professional expertise also change the processing of components involved (Mulas et al., 2012).

### Personality and Aesthetic Sensitivity

Regarding variation in human experience, inner states, and behaviors, personality as traits (i.e., rather stable attributes) mark habitual patterns of behavior, thought, and emotion. Despite disagreement among researchers regarding the number nor the attributes of dimensions and personality facets, there are some popular inventories to capture interpersonal differences; sometimes researchers include personality inventories such as the Big Five in order to detect non-trivial relationships, often in an exploratory fashion. Regarding preference of complex images of artworks, Furnham and Walker (2001) showed a positive correlation of Neuroticism with preference for abstract and pop art, while Conscientiousness, another dimension of the big five, correlated with preference for representational paintings. Moreover, Openness to Experience was associated with a general liking of all art. Lyssenko et al. (2016) found that participants with higher scores for Neuroticism showed preferences for objectively more complex images when compared with participants with lower scores for Neuroticism. Kim and Kim (2019) associated Big Five user profiles with color use (on Instagram) and identified complex color relations with agreeableness, gender, loneliness, or extraversion. There are now various studies aiming to correlate photos in social media with personality (Lay and Ferwerda, 2018; Cooper et al., 2020; Rodriguez et al., 2021) but these reveal little about the features that determine experienced beauty of MPP. Thus, it is still an open question how these kinds of findings can be informative regarding beauty and aesthetic appeal of MPP.

### Gender Differences

Not much is known about the effect of gender on beauty in images. Surely, an individual’s gender-role identity involves more than simply recognizing oneself as male or female (O’Brien, 1992). Thus, studies of gender presumably will be interesting when they associate gender with certain general behavior in using MPP for different tasks that also vary in meaningful ways with gender, for example, as in mating, or images of more gender-biased products, or in evolutionary gender roles. To provide one example regarding color preferences, Hurlbert and Ling (2007) revealed a sex difference in the weighted “red-green” cone contrast: females preferred “reddish,” and males preferred “greenish” contrast against the background, and the authors speculated that evolutionary gender roles of females as fruit-gatherers (red fruit against green foliage) could explain their preference.

### Developmental Aspects and Age Differences

Age has effects on preferred images at least on two levels. First, age comes along with periods of growth and change, in which preferences develop due to youth culture and peer agreement, and taste is acquired through familiarity. Thus, it can be assumed that belongingness to a historical period has a strong effect on what people like. Empirical aesthetics has its limitation of being very presence-focused, as perceivers of images are usually all tested at the same time, usually using the same kind of material. However, individually assessing familiarity with media and purposes of use should distinguish different peer and age groups. Second, psychologically there are of course changes with aging, as certain aspects of aesthetics develop with aging, as revealed by Schabmann et al. (2015) who measured evaluations of perceived beauty, and other evaluations for school children of different age. In all conditions, structural equation models revealed significant effects of emotion and the dependencies between emotion and liking (Leder et al., 2004) were consistently higher for the younger children. On the other hand, the interactions between arousal and liking, as well as understanding, were higher for older school children. These results indicate a transition from an affective toward an increasingly cognitive knowledge-based sense of aesthetics, and generally provide evidence for the fundamental importance of emotional processing. Another aspect of aging has been addressed by Orzech et al. (2017) who distinguished between a more “inwards oriented perspective” of young adults in contrast to a more outward oriented use of images by older people, representing differences in digital social norms of co-creation of self, as well as a priority for privacy for others. The relations to self-image was also stressed by Pelowski et al. (2017) as an important feature to understand aesthetic responses.

The human visual system also changes with age. Regarding very early developmental stages and the differentiation between “color” and “brightness” (Specker et al., 2018), the pathways for achromatic neural responses appear to be stable in infants already after a few months of age (Crognale, 2002) while chromatic neural responses continue to develop from birth until puberty (Boon et al., 2007), so that both pathways age differently. Moreover, these studies may indicate “that the chromatic system may be more sensitive to cultural influence than the achromatic system.” (Specker et al., 2018, p. 48). Regarding color preferences, Skelton and Franklin (2020) also argued that infant looking behavior, as well as adult color preferences, are at least partially rooted in the sensory mechanisms of color vision. Especially with older age, there are systematic changes in the physiology of the eye that affect how images are perceived but we could assume that the changes due to historic context-dependent “image cultures” (Redi et al., 2016) might have a stronger influence on aesthetic preferences compared to physiological aging effects. Differences in digital affinity and peer-group effects might also exert stronger influences than research so far revealed. However, such effects are probably particularly sensitive to changing fashion trends and fast developing technical innovations, which make generalizations beyond specific historical periods rather difficult.

## Cultural Effects

Users of MPP also differ in respect to cultural influences. There is a permanent quest in empirical aesthetics for universally shared features (see Che et al., 2018), such as some of the visual features discussed in early processing, and research tries to clarify if they are the basis for fundamental perceptual and valuation processes that are shared by all humans, such as preferences for symmetry or curvature for which Gómez-Puerto et al. (2018) showed that participants from Ghana, Spain, and Mexico all showed a trend to prefer curved stimuli. Cultural differences can be observed from early visual features to higher order criteria of beauty specific to certain object genres, and these differences should not be neglected in a comprehensive research program on the factors that determine the perceived beauty of images.

Regarding low-level visual image features, van Dongen and Zijlmans (2017) reported empirical support against the universal importance of contrast for painting, but in favor of the universal importance of contrast in relation to images of people characteristics. Al-Rasheed (2015) found that Arab and English individuals showed different hue preferences, yet there was greater similarity for Arab and English males than Arab and English females. Saito (1996) found for three Asian regions, that each had unique color preference tendencies, and all had a high preference for the color white that, besides the factors of age and sex, probably is due to associations based on environmental and cultural environment. This is in accordance with Taylor et al. (2013) who argued against universal color preferences, based on an empirical comparison between British and Himba participants. They studied cone contrast, object associations, and colorfulness, and found that “the relationship of these predictors to color preference was strikingly different for the two cultures” (p. 1015). To study color and emotion associations, Jonauskaite et al. (2020) tested more than 4,500 people from 30 nations and found evidence for some universality, but also variations that could be explained by linguistic and geographic similarities. In faces, there is cultural variation in preferences for skin color. As Dixon and Telles (2017) explained, for women in particular, lightness has been associated not just with the leisure class, but that “in Japan, whiteness has for centuries, if not more than a millennium, held symbolic meanings and associations with class privilege, spiritual purity, and feminine beauty.” Changing skin toward white is not popular in Europe, but in Nigeria, South Africa, and Togo, 77, 35, and 59% of women, respectively, regularly use skin-lightening products. In China, Malaysia, the Philippines, and South Korea, approximately 40% of women use such products (WHO/PAHO, 2012). The effect of skin tone on attractiveness was also studied by Hill (2002) who found skin tone influences the attractiveness ratings assigned to black women in a compelling, monotonic manner—the fairer the tone the more attractive. Similarly, Ip et al. (2019) further showed that, for Caucasian and Chinese participants, higher levels of skin carotenoid coloration (reddishness) were preferred in face and body parts, but not in non-face (scrambled pattern) stimuli. While Coetzee et al. (2014) found some support for cross-cultural agreement, but also, that south-African participants relied more on skin color, and Scottish participants more on face shape features. Similarly, Han et al. (2018) showed cultural differences, as an interaction of preference for yellowness depending on cultural origin of the rater; as well as reduced preference of Chinese participants for facial redness and a stronger preference for facial lightness compared with United Kingdom participants. All these differences challenge the idea of universal standards of face beauty.

There are also other differences regarding faces, for example in selfies. Huang and Park (2013) reported evidence for the often-assumed more contextual Asian, versus more object-centric western cultures, by showing that East Asian Facebook users were more likely to deemphasize their face and include more background context in their profile pictures, while Americans tended to prioritize their focal face and exposed more intensity of facial expression, but included less background. There are also approaches that aim to analyze variation based on demographic background through deep convolutional neural networks (e.g., Kairanbay et al., 2019). Thus, it should be worthwhile to investigate how criteria for beauty might differ across cultures. Climatic, nutrition or religious differences could affect what is liked in images, and what—in critical cases—condemns certain kinds of images completely (see Freedberg, 1991, on iconoclasm). Several studies showed that beauty ideals vary across cultures and these cultural differences correlate with variables beyond what is usually associated with beauty, such as the “health of a nation” (DeBruine et al., 2010). For mobile phone cameras, which are used by a large part of the population all over the world, the different sources of what is considered beautiful require more research efforts, as the variation between cultures is a still underexplored topic in aesthetic research.

## The Purpose: Motivation, Goals, and Situational Functions

To close the circle, we return to the different tasks, and functions of images, and more specifically, MPP. From fast swiping in a sequence of similar images, to deep aesthetic pleasure when indulging in images of one’s own wedding, or vacation, image beauty varies with task, purposes, and functions. A full taxonomy of tasks that people engage in when dealing with MPP is yet to be established. However, we can make some assumptions based on which kinds of images have the highest prevalence in MPP use. Hu et al. (2014) distinguished the most frequent classes of objects, and also identified five main profiles of users. Of course, these findings are descriptive and purely data-driven, but together they allow for some assumption regarding broader classes of motifs, goals, and functions.

Main motivations and goals can be either specific for how images are used that have been taken with mobile phone cameras or are rooted more in personal motifs of the user (Hu et al., 2014). Examples for the former are communication (see communicative purposes, Stefanone et al., 2010) *via* social networks, and preserving visual memories (Kuhn, 2010), or to document an event, or scanning a document, or even producing a photographic artwork. Examples for the latter might be more personally rooted, such as designing a nostalgic, or actual narrative of one’s life, or aim for finding a partner or friend in a social network (Schwarz, 2010). More systematically, Kocak et al. (2020) identified six main usage motive categories which they named as self-expression, recording, socialization, recreation, creativity, and prying. Lee et al. (2015) analyzed Instagram users, and identified five primary social and psychological motifs: social interaction, archiving, self-expression, escapism, and peeking. Sung et al. (2016) found four motivations for posting selfies on social networks, which are attention seeking, communication, archiving, and entertainment. Thus, for different classes of images, certain typical motivations, goals and functions can be distinguished, to which beauty could contribute differentially.

We discussed various components that affect the beauty of images taken with mobile phones. We have largely ignored computational approaches to finding correlates between beauty and mathematical or pixel-based analyses of images (a more in-depth overview on this topic can be found in Brachmann and Redies, 2017). In special cases, images of individually meaningful events can elicit very strong emotions (e.g., positive such as marriage and love, negative such as war and loss, or sad such as images of a lost person), and can become very important objects, which is usually highly idiosyncratic, but occasionally even shared and collective (see for example the visual nature of flashbulb memories, Lanciano et al., 2018). Other specifically strong responses which occur only in some viewers and were not discussed in our paper are phenomena such as spider fear, or prosopagnosia, which affect the perception of specific topics; or even religious or ideological, political images that elicit emotions, and aesthetic admiration—or the opposite. Also, erotic images are made to elicit specific kinds of arousal, and taboos can result in the strongest rejections, similar to the more fundamental state of iconoclasm. Due to limited space we omitted a discussion of papers that already tested some interactions between variables that we discussed (e.g., Tinio and Leder, 2009b; Little, 2014; Oren et al., 2020).

## Conclusion

We proposed a foundation for systematic research into the psychological and contextual variables that contribute to experienced beauty in MPP. We hope to inspire research toward a wholistic understanding of the factors determining the aesthetic quality of MPP and other fields of photography. Our ideas could find applications in computational modeling, to improve predictions of aesthetic preferences by taking a broader set of user-related and contextual variables into account. This comprehensive approach to understanding the aesthetic appeal of MPP can also lay ground for developing imaging technology that tailors the produced images to the individual users and their goals. This could not only maximize the happiness with the devices and the pictures that they produce but also enrich the life of the users through outstanding aesthetic experiences that can be shared with other people.

## Author Contributions

HL wrote the first draft of the manuscript. All authors contributed to the article and approved the submitted version.

## Funding

This project received funding from Huawei Technologies Oy (Finland) Co. Ltd. The open access publication is supported by the University of Vienna.

## Conflict of Interest

JH and V-TP are employed by Huawei Technologies Oy (Finland) Co. Ltd. This project received funding from Huawei Technologies Oy (Finland) Co. Ltd. who internally approved the decision to publish the manuscript.

The remaining authors declare that the research was conducted in the absence of any commercial or financial relationships that could be construed as a potential conflict of interest.

## Publisher’s Note

All claims expressed in this article are solely those of the authors and do not necessarily represent those of their affiliated organizations, or those of the publisher, the editors and the reviewers. Any product that may be evaluated in this article, or claim that may be made by its manufacturer, is not guaranteed or endorsed by the publisher.

## Footnotes

^1^https://focus.mylio.com/tech-today/how-many-photos-will-be-taken-in-2020; https://www.statista.com/statistics/330695/number-of-smartphone-users-worldwide/

^2^Note, this paper does take into account, to some extent, important ideas and work from humanities, art history (e.g., Gombrich, 1960; Freedberg, 1991), and image sciences in the humanities. However, to fully review these would exceed the scope of this project, whereas the focus on empirical studies in aesthetics allows us to better gear the theoretical work and its result to ongoing empirical research.

^3^ https://kunstgeschichte.univie.ac.at/forschungsprojekte/crea/time-makes-the-difference/
